# Precision and Accuracy of Receptor Quantification on Synthetic and Biological Surfaces Using DNA-PAINT

**DOI:** 10.1021/acssensors.2c01736

**Published:** 2023-01-19

**Authors:** Roger Riera, Emmanouil Archontakis, Glenn Cremers, Tom de Greef, Peter Zijlstra, Lorenzo Albertazzi

**Affiliations:** † Department of Biomedical Engineering, Institute for Complex Molecular Systems (ICMS), Eindhoven University of Technology, P.O. Box 513, Eindhoven5600 MB, Netherlands; ‡ Laboratory of Chemical Biology and Institute for Complex Molecular Systems, Eindhoven University of Technology, P.O. Box 513, Eindhoven5600 MB, The Netherlands; § Computational Biology Group, Department of Biomedical Engineering, Eindhoven University of Technology, P.O. Box 513, Eindhoven5600 MB, The Netherlands; ∥ Institute for Molecules and Materials, Radboud University, Heyendaalseweg 135, AJ Nijmegen6525, The Netherlands; ⊥ Department of Applied Physics and Institute for Complex Molecular Systems, Eindhoven University of Technology, P.O. Box 513, Eindhoven5600 MB, The Netherlands; # Nanoscopy for Nanomedicine, Institute for Bioengineering of Catalonia, Barcelona08028, Spain

**Keywords:** DNA-PAINT, single-molecule, super-resolution microscopy, biosensors, receptors, quantification

## Abstract

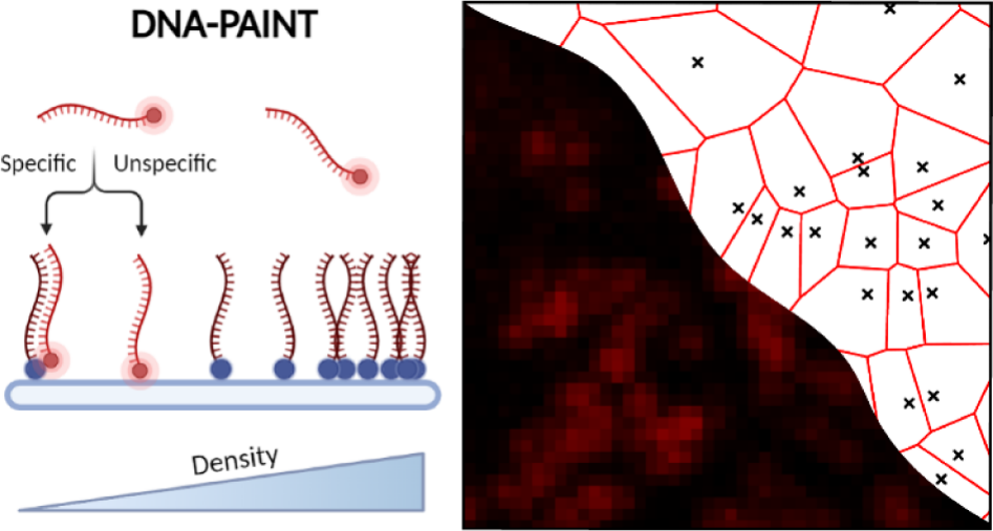

Characterization of the number and distribution of biological molecules on 2D surfaces is of foremost importance in biology and biomedicine. Synthetic surfaces bearing recognition motifs are a cornerstone of biosensors, while receptors on the cell surface are critical/vital targets for the treatment of diseases. However, the techniques used to quantify their abundance are qualitative or semi-quantitative and usually lack sensitivity, accuracy, or precision. Detailed herein a simple and versatile workflow based on super-resolution microscopy (DNA-PAINT) was standardized to improve the quantification of the density and distribution of molecules on synthetic substrates and cell membranes. A detailed analysis of accuracy and precision of receptor quantification is presented, based on simulated and experimental data. We demonstrate enhanced accuracy and sensitivity by filtering out non-specific interactions and artifacts. While optimizing the workflow to provide faithful counting over a broad range of receptor densities. We validated the workflow by specifically quantifying the density of docking strands on a synthetic sensor surface and the densities of PD1 and EGF receptors (EGFR) on two cellular models.

Quantitative analytical tools to measure (bio)molecular concentrations are of foremost importance in fundamental, clinical, and industrial research. Although there are many techniques to measure the concentration of molecules in solution,^1^ it becomes more complicated when they are bound to a surface.^2,3^ This presents similar issues for both synthetic surfaces (i.e., biosensors) or biological surfaces (i.e., cell membranes). The density and distribution of molecules on surfaces are critically important for the interactions with the local environment: for instance, how a biosensor interacts with its analyte or a cell with a therapeutic agent.^4^ Moreover, proteins on the surface of the cells, such as receptors, are not only important because of their interactions with therapeutics, but they are also used as predictive biomarkers for diagnostics.^5,6^

Most of the available techniques to quantify the density of molecules on surfaces tend to lack sensitivity and do not give information about the distribution. For instance, classical optical microscopy lacks the resolution to distinguish nearby molecules at the nanoscale.^7^ Flow cytometry does not reveal spatial information, or ensemble measurements, such as ELISA, require the separation of the target molecule from the surface into the solution (i.e., cell lysate). Therefore, a sensitive method to quantify the density and measure the distribution of molecules on surfaces with accuracy and precision is imperative.

In the last decade, super-resolution fluorescence techniques that overcome the resolution limitation of conventional fluorescence microscopy have enabled fluorescence imaging at the nanoscale in many fields.^8^ These techniques not only improve the resolving potential for structures below the diffraction limit but also can be used as powerful quantitative tools.^9^ For instance, single-molecule photobleaching measures the photobleaching steps of a fluorescently labeled sample to determine the number of molecules on a synthetic surface with each bleaching step representing a single-molecule. However, it is limited to low density samples and thus is vulnerable to noise.^10,11^ Alternatively, balanced SOFI analysis which relies on photo-switchable dyes can be used to count discrete molecules on a surface.^12^ However, it is limited to low emitter densities and single-molecule photo-switching rate variation. Intensity-based methods (e.g., IBC) can provide information about the number of labels and the amount of molecules but still requires an intensity standard and decent flat-top type illumination for homogeneous excitation.^13^ Recently, examples of quantitative single-molecule localization microscopy in cell biology^9^ and in synthetic materials^14^ have emerged. Due to the nanometric spatial resolution and single molecule sensitivity, these are promising tools to quantify the number and distribution of receptors in biosensors,^15−17^ cells,^18−20^ and extracellular vesicles.^21,22^

DNA-PAINT (Point Accumulation for Imaging in Nanoscale Topography) has become one of the quantitative methods for single-molecule localization microscopy (SMLM). It is based on the short transient interaction of a labeled DNA probe (imager) to a complementary DNA target molecule (docking).^23^ The bound time of the DNA–DNA interaction is highly dependent on the length DNA duplex that is formed and can be optimized by tuning the length base pair (bp) leading to typical bound times in the order of milliseconds or seconds and independent of the imager concentration.^24^ Therefore, only a fraction of the target molecules is localized at a time, and the process is repeated over time to produce a complete reconstructed image of all target molecules. The quantitative properties of DNA-PAINT are superior to other SMLM techniques since the quantification is based on DNA interaction kinetics rather than photophysical properties such as the stochastic blinking of dyes.^25^ Recent experiments in well-controlled systems based on DNA origami showed the potential of quantitative PAINT and fluorescence correlation spectroscopy (FCS) to achieve molecular counting.^26^ Pioneering studies then applied DNA-PAINT to image cellular structures^27^ and biomaterials.^17^ However, the methods are still far from standardization. For instance, the quantification of single molecules on a surface with DNA-PAINT can be achieved by identifying single sparse emitters using their spatial information—repeated binding events would create dense spots that can be identified using multiple clustering algorithms^9,28^ —or through the expected kinetics of the specific DNA binding sequence (qPAINT), but there is no objective assessment of which is more adequate. Moreover, the experimental conditions such as imager concentration, imaging area, and imaging duration as well as the analysis workflows are often empirically chosen because the interplay between imaging conditions, quantification accuracy, and precision are variable.

Here, a standardized DNA-PAINT workflow is presented to quantify density and distribution of molecules on surfaces, by finding the optimal acquisition parameters and tailored filtering of non-specific interactions to maximize precision and accuracy. In this work, the following considerations were addressed: (i) which is the most adequate DNA-PAINT quantification approach based on molecular density; (ii) what are the optimal conditions to obtain the fastest, more accurate, and precise qPAINT measurement, and (iii) non-specific localization filtering on complex substrates such as cell membranes. With the help of simulations and experiments, the optimum experimental conditions were determined for diverse samples to offer a robust quantification over a broad range of molecular densities. First, the workflow was applied to a controlled in vitro environment by functionalizing a glass surface with DNA strands with controllable varying densities. The model was used as a synthetic surface, such the one found on a biosensor, which allowed for elucidating the effect of imaging parameters for the optimal quantification precision. Finally, these insights were applied in combination with a non-specific localization filtering to quantify the density and distribution of membrane receptors on different cell lines, while keeping a high precision and accuracy.

This work provides guidelines to perform quantitative measurements on surfaces, providing single-molecule sensitivity, while at the same time offering means to optimize precision and accuracy over a broad range of molecular densities. Routine usage of super-resolution microscopy for the quantification of surfaces will provide a route to tailored synthetic surfaces and provide a tool to quantify molecular distributions on cell membranes.

## Results and Discussion

In order to standardize and objectively select the optimal acquisition and analysis of DNA-PAINT data, it is vital to introduce a means to assess the robustness of a counting method by two metrics: (i) the counting precision which indicates the spread of the counted number of molecules (could be a DNA strand on a glass surface or a membrane receptor) when repeated measurements are performed and (ii) counting accuracy, which denotes to what degree the mean number of counted molecules deviates from the true number (the ground truth). To investigate these two metrics, experiments were first performed on DNA-functionalized glass slides. This approach allows for quantifying the density and distribution of docking strands at multiple molecular densities in a controlled manner by varying the functionalization conditions.

**Figure 1:**
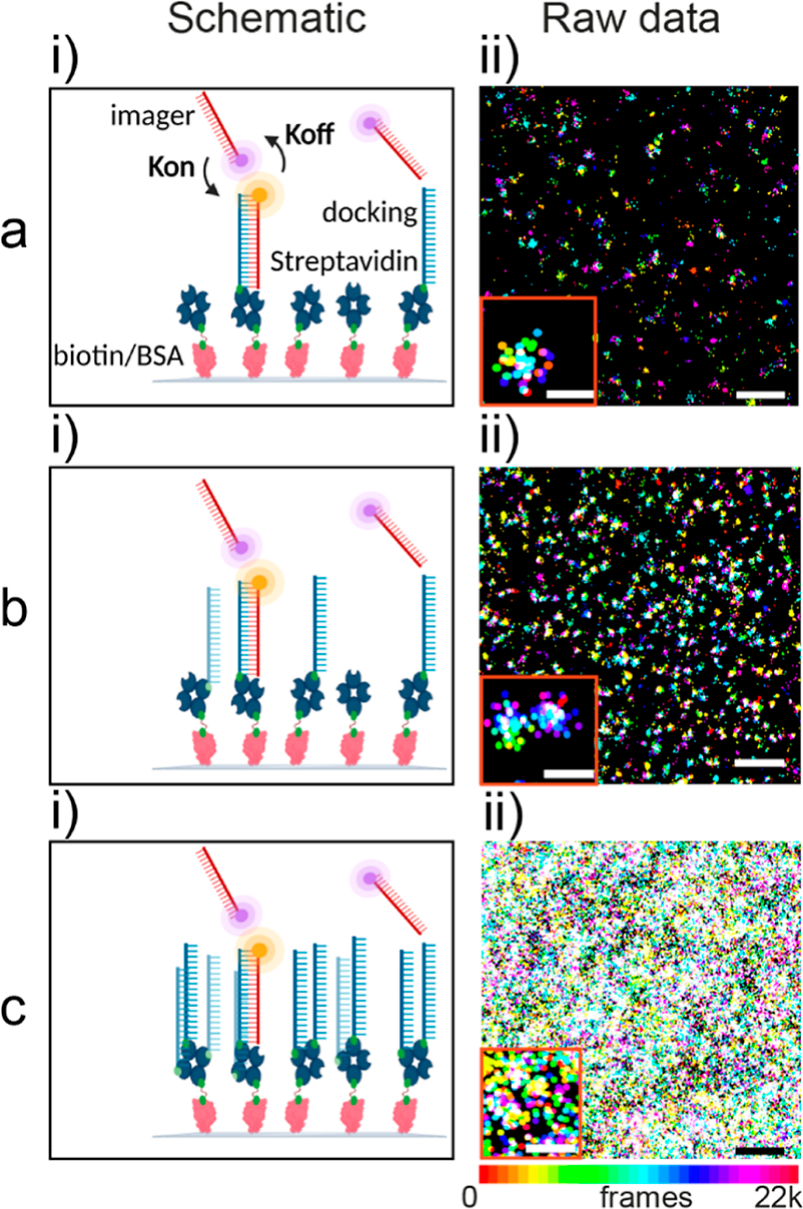
Schematic representation of the DNA-PAINT workflow on glass slides. a(i), b(i), and c(i) show the sample geometry with different concentrations of biotinylated DNA docking strands (blue), conjugated to a glass surface through fixed BSA-biotin (red-green) and streptavidin (blue) passivation. Fluorescently labeled (ATTO 655) imager strands (red) transiently bind to their complementary docking sequences. The resulting fluorescent bursts are localized using a Gaussian fitting, resulting in the reconstructed images in the right. The datapoints are colored based on the camera frame in which the event occurred. [a(ii), b(ii), and c(ii)] DNA-PAINT reconstructions for three different docking densities; low (2.5 nM), intermediate (12.5 nM), and high (30 nM), respectively. The insets show a magnification of a small area. Scale bar: PAINT images (200 nm), PAINT insets (50 nm).

Figure 1 highlights schematically the single-molecule DNA-PAINT measurements on DNA-functionalized glass slides. First, a BSA-biotin-streptavidin antifouling coating is used as the support to functionalize our slides with biotinylated DNA docking strands (Table 1) at different concentrations—low, intermediate, and high—as shown in Figure 1a–c (more densities are displayed in Figure S1). This biotin-streptavidin-based functionalization allows us to access a wide range of densities in a controlled fashion. Subsequently, the complementary fluorophore-labeled DNA imager strand is added to the solution. Transient hybridization of the imager to the docking strands generates diffraction-limited fluorescence bursts that are detected across thousands of frames in a single movie (Figure S2a). These fluorescent bursts or PSFs (point-spread functions) are fitted with a Gaussian function to obtain the center of the diffracted-limited spot and precisely determine the position of the target molecule. The merging of all single-molecule positions results in super-resolved reconstruction maps as illustrated in Figure 1a(ii),b(ii),c(ii), for the low, intermediate, and high-density samples, respectively. As expected, increasing the DNA docking concentration added to the slides resulted in an increased number of localizations in the DNA-PAINT reconstructed images, which reflected the higher amount of docking strands bound to the glass.

**Table 1:** DNA Imager and Docking Sequences

name	docking sequence	supplier	imager sequence	supplier	experiment
Sequence 1 (9mer) ATTO655	Biotin- TTA TAC ATC TA	IDT	CTA GAT GTA T—ATTO655	IDT	Figures 1,2 and 3 and S1,S2 and S4
Sequence 1 (10mer) ATTO655	NH2- TTA TAC ATC TAG	IDT	CTA GAT GTA T—ATTO655	Eurofins	Figure S6
Sequence 1 (9mer) ATTO647N	NH2- TTA TAC ATC TA	IDT	CTA GAT GTA T—ATTO647N	IDT	Figure S5
Sequence 1 (10mer) ATTO647N	NH2- TTA TAC ATC TAG	IDT	CTA GAT GTA T—ATTO647N	IDT	Figures 5 and S5
Sequence 2 ATTO647N	-	-	TAT GTA GAT C—ATTO647N	IDT	Figure S5
Sequence 2 ATTO655	-	-	TAT GTA GAT C—ATTO655	IDT	Figure S4

Figure 1a(ii) illustrates a reconstructed image that contains the retrieved localizations from a low-density sample (concentration of 2.5 nM). Multiple points occurring stochastically throughout the imaging time at a specific spot [multicolor clustered localizations, inset in Figure 1a(ii)] were observed, which correspond to the specific binding events on a single isolated docking strand. Increasing the density of docking strands (by increasing the docking concentration to 12.5 nM during the substrate functionalization) results in an increased number of isolated clusters as seen in Figure 1b(ii) (multicolor clustered localizations in the inset). However, in Figure 1c(ii), which highlights a higher docking density (concentration of 30 nM), these clusters cannot longer be observed since they spatially overlap due to their distance, which is lower than their actual size (determined by the localization precision of the measurement). There are two approaches to analyzing and quantifying these data: (i) identifying single molecules by the resulting spatial clustering of localizations due to repeated binding events (here referred as direct counting), generally through a clustering algorithm, and (ii) extracting the molecular count from the expected binding kinetics of the DNA pair (docking imager), known as qPAINT^25^ (here referred to as kinetic counting). In the next section, the performance of the two counting approaches is shown in terms of their precision and accuracy across the broad range of synthetic surface densities visualized in Figure 1.

### Direct Counting on Synthetic Surfaces with Mean-Shift Clustering

**Figure 2:**
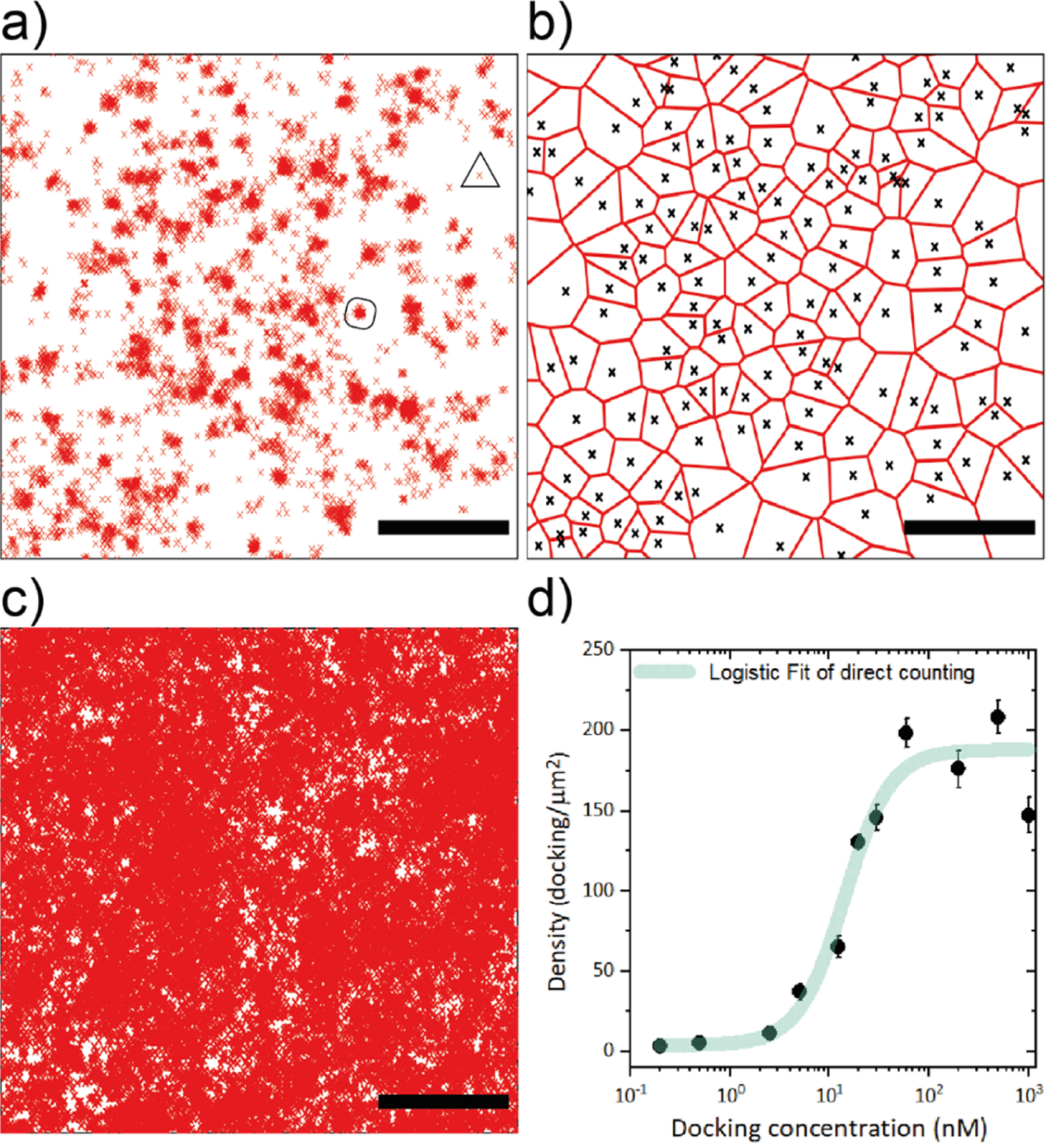
Direct counting of DNA docking strand receptors on glass slides by mean-shift clustering. (a) Reconstructed PAINT localizations (red points) of a 12.5 nM docking slide, (b) Voronoi tessellation after clustering analysis displaying the density and distribution (black crosses denote single clusters representing single docking strands). (c) Reconstructed PAINT localizations of a 30 nM docking slide, where it is not possible to distinguish single receptors. (d) Density of DNA docking strands retrieved with direct counting, where model surfaces were prepared by exposing BSA/biotin/streptavidin-coated glass slides to increasing concentrations of biotinylated docking strands (black dots represent mean ± σ of eighteen counting areas as depicted in a–c). Logistic equation fit (transparent line) yields a mid-point concentration of 14 ± 2 nM; a slope of 1.8 and *R*^2^ of 0.98. Scale bar: PAINT images (500 nm).

Currently, there are several image-processing methods able to identify and characterize clusters of localizations.^28^ These kinds of approaches require molecules sparse enough to prevent spatial overlap of the localizations that belong to neighboring clusters. This means that, in practice, the clusters (i.e., the docking strands) should be separated by more than the spatial localization precision of the microscope (Figure 2a). If this is the case, information about the ground truth position and spatial distribution can be extracted, which is of crucial importance for synthetic and biological surfaces.^29^ Here, a custom MATLAB algorithm was employed (see Methods section for detailed description), based on mean-shift clustering, to group localizations into clusters using a pre-specified cluster size. This clustering algorithm was chosen since it has been used extensively in single-molecule localization microscopy data to identify single molecules and nanoparticles and does not require a priori knowledge of the amount of clusters.^28,30^ In this procedure, non-specific interactions are routinely discarded because they appear as sparse single points on the coverslip and do not belong to a cluster (black triangle in Figure 2a). Therefore, after filtering out the sparse points, only clusters of a certain size (50 nm), which have a minimum number of events are selected (black circle in Figure 2a). This was assessed with control measurements to show that approximately only 15% of the clusters are non-specific, thanks to the antifouling BSA coating (Figure S4a). Then, the number of specific clusters (docking strands) per surface area is counted to obtain the average density as well as a Voronoi tessellation was used to visualize the spatial distribution on the coverslip (Figure 2b).

This approach quantifies the density of docking strands, which is varied by exposing the substrate to increasing concentrations during the functionalization step, ranging from 0.2 nM to 1 μM (Figure 2d). Clusters appeared adequately separated at docking concentrations below 15 nM and were directly counted showing an average of 3 to 65 molecules/μm^2^ from 0.2 to 15 nM of docking, respectively. Notably, across these conditions, the number of binding events per cluster is comparable and follows the expected Poisson statistics, confirming that each identified cluster indeed represents a single docking strand (Figure S2e). Hence, the direct counting approach on the synthetic surface allowed for identification and localization of receptors with single-molecule sensitivity at low surface densities.

Conversely, when the substrate is incubated using concentrations above 20 nM, docking strands are separated by less than the localization precision of our technique and the reconstructed images start to saturate (Figure 2c). Although the density of docking strands increases with docking concentration, their counted density using clustering does not increase further (Figure 2d). This depicts a upper and lower bound of the counting range of the method of around 190 ± 15 and 4 ± 3 molecules/μm^2^ (density ± SE), as extracted from a logistic fitting in Figure 2d. Moreover, the mid-point docking concentration of the method is 14 ± 2 nM, which corresponds to 95 ± 2 molecules/μm^2^ The upper bound level denotes a systematic undercounting of the direct counting method when used on high docking concentrations (>20 nM) with the higher density counts lying into the upper bound level. Therefore, the counting is no longer increasing with increasing docking concentration (Figure 2d). To confirm this finding, clusters at different densities were simulated to assess the accuracy of the counting methodology. Consistent with the experimental data, the accuracy starts to drop notably between 50 and 100 molecules/μm^2^, setting a limit for the direct counting at higher densities (Figure S3a).

Hence, direct counting is capable of accurately localizing and counting individual receptors at molecular densities below 100 molecules/μm^2^ providing single-molecule sensitivity while ignoring non-specific interactions based on the absence of repeated localizations on the same location. Since single molecules are directly observed, the variability error from the method is minimal offering extreme precision. However, the method has limited applicability for higher molecular densities, for which kinetic counting is preferable as shown in the following section.

### Kinetic Counting on Synthetic Surfaces with qPAINT

At high molecular densities, dockings are densely packed. This inevitably leads to spatial overlap of single clusters; thus, direct counting approach is no longer feasible to be applied. Therefore, we applied a statistical approach (namely qPAINT^25^), in which the distribution of times between binding events (dark times) is measured to extract the total number of docking strands (*n*) in a surface area (*A*). As an example, the fluorescence time trace of a typical field of view covered with dockings is shown in Figure S2. The fluorescence bursts correspond to binding events, from which we can extract the dark times in between those events. With the fitted average value of dark times (τ_d_), we can obtain the number of dockings in that area using eq 1 (see Methods section). A calibration of the k_on_ value of the specific docking–imaging pair is needed in the equation, which we obtained from the distribution of dark times of individual clusters on a low-density sample (Figure S4b). It yielded a *k*_on_ = 1.31 × 10^6^ M^–1^s^–1^, in good agreement with previously reported values.^17,24^

**Figure 3:**
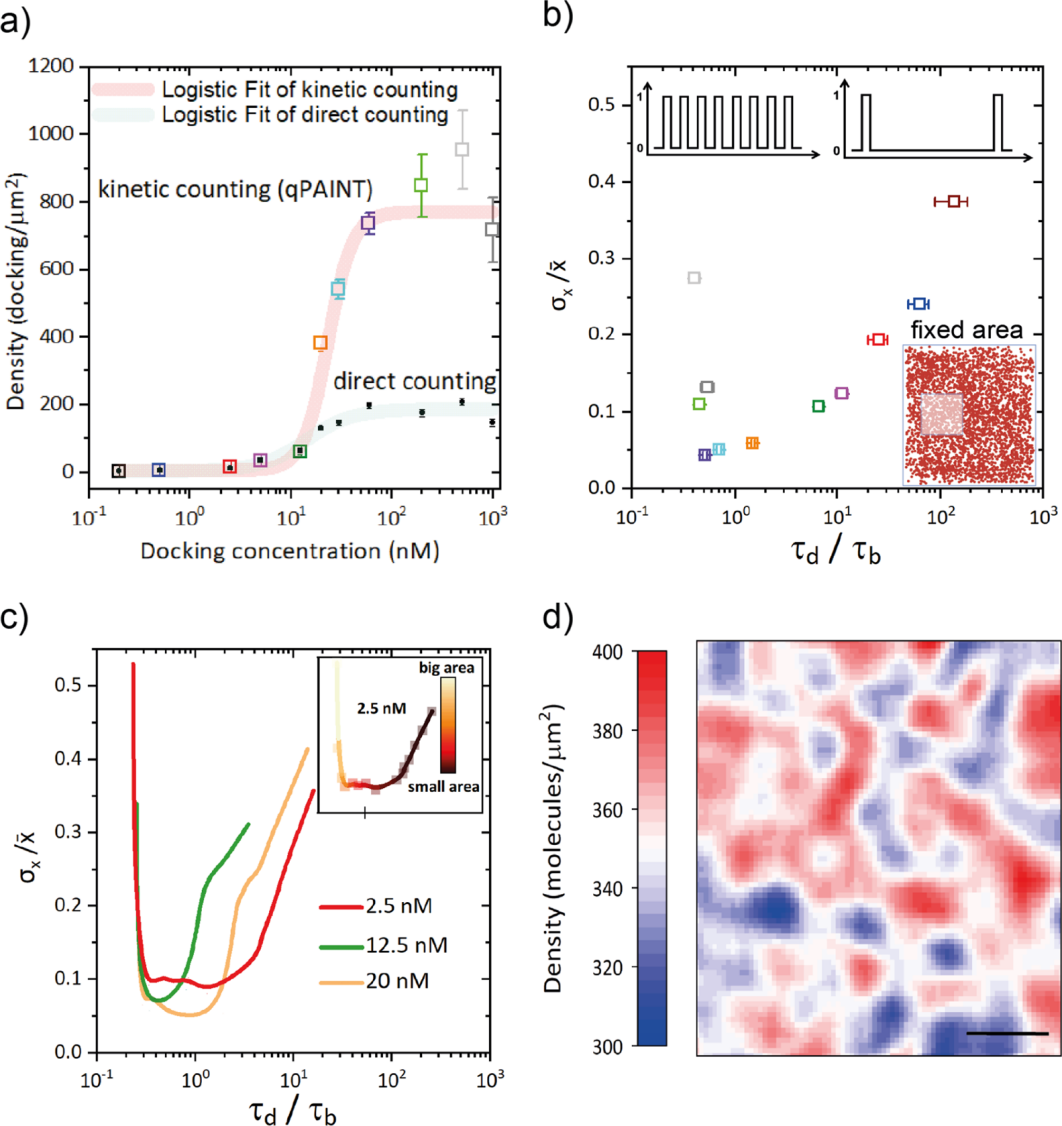
Kinetic counting of receptors on glass. (a) Comparison of the number of single docking sites per area retrieved with direct counting (black dots from Figure 2) and qPAINT (colorful squares), on the same raw data (mean ± σ). Logistic equation fit (transparent line) yields a mid-point concentration of 23 ± 1 nM, a slope of 3.5, and an R^2^ of 0.94. (b) Experimental counting precision of qPAINT for the different docking concentrations, as a function of the ratio of dark over bright times, using a fixed counting area of 1 μm^2^ (mean ± σ). Each colored dot corresponds to the same docking incubation concentration from a. (c) Experimental counting precision for three docking concentrations (2.5, 12.5, and 20 nM), as a function of τ_d_/τ_b_ by varying the counting area from smaller to bigger three. The three docking slides (2.5, 12.5, and 20 nM) were imaged with 2.5 nM imager concentration τ_d_/τ_b_ for 2.5 nM docking. The color (Inset) of a single precision graph versus τ_d_/τ_b_ for the 2.5 nM docking slide. The colored line grading depicts the varying size of the counting areas for the given docking concentration (2.5 nM). (d) Docking density distribution (30 nM) on the glass slide measured with qPAINT. Scale bar: 2 μm. Receptor imaging and quantification on the membrane of cells.

In this way, the set of docking functionalized slides was assessed. It was observed that at the point that the direct counting approach saturated due to overlapping of clusters, the qPAINT showed a response to increasing docking concentration beyond that point. This is visualized in Figure 3a, where kinetic counting keeps its linear dependency in a much broader range of docking concentrations. As the graph shows, for concentrations above 15 nM, we counted almost a 3-fold higher docking density compared to direct counting. For instance, the 30 nM docking slide yielded approximately 180 molecules/μm^2^ using the direct counting approach (which showed previously that there was cluster overlapping), while with the kinetic approach yields 554 molecules/μm2. In order to assess the counting dynamic range of the kinetic method, a logistic fit was applied on the kinetic data sets (Figure 3a). This leads to a lower bound of 6 ± 3 molecules/μm2, higher bound of 771 ± 112 molecules/μm^2^, and a mid-point docking concentration at 23 ± 3 nM, which corresponds to 386 ± 3 molecules/μm^2^ (density ± SE). This corroborates the clear undercounting of the clustering approach at higher molecular densities and confirms that the kinetic approach extends the dynamic range of counting for high concentrations (>20 nM). This result proves the high accuracy of qPAINT in counting the exact number of receptors on 2D surfaces for a broad range of densities. In theory, the qPAINT approach can be applied to any density range and obtain the same precision and accuracy, if the average dark time (τ_d_) is accurately retrievable. This depends on the number of dark times used to extract this value; therefore, we calculated the accuracy and precision of qPAINT on the simulated time traces with different statistics (Figure S3b). While it is observable that the accuracy reaches a plateau quite quickly (above 10 dark times), the precision seems optimum from 100 dark times onward. Therefore, a minimum of 100 dark times was set as the target for highly accurate and precise qPAINT measurements.

Although qPAINT performance is mainly influenced by statistics and could be applied at any density range, we observe that the density calculations also reach a plateau and display higher variability in the slides above 100 nM docking (note the error bars in Figure 3a). This is because all experiments were performed using a constant area (*A*) and imager concentration (*c*_i_), resulting in temporal overlap of binding events at higher docking densities. This is easily circumvented by reducing *A* or *c*_i_,^25^ so we decided to explore which are the optimal conditions to achieve precise counting. To evaluate the counting precision, the ratio of dark and bright time (τ_d_/τ_b_) was tuned in two ways: (i) fixed *c*_i_ and *A* at different docking concentrations (Figure 3b) and (ii) fixed *c*_i_ and docking concentration with varying *A* (Figure 3c). The precision was plotted as a coefficient of variation, that is, the standard deviation of the count divided by its mean, obtained from repeated experiments on the same sample. In both cases, a similar behavior is observed; an optimal τ_d_/τ_b_ ratio around 1 maximizes precision, which is in agreement with previous studies.^16^ In Figure 3b, a lower ratio (τ_d_/τ_b_ < 1) shows a higher coefficient of variation because binding events overlap in time, resulting in undercounting. Similarly, a higher ratio (τ_d_/τ_b_ > 1) also shows a higher coefficient of variation, in response to a lower amount of events collected (Figure S3b). This was evaluated in a different way in Figure 3c, where instead of varying the concentrations to tune the τ_d_/τ_b_, we varied only the counting area on a given docking concentration. The graph corresponds to τ_d_/τ_b_ values at different counting areas of three docking concentrations from Figure 3b. They all follow the same trend with a maximum precision at an approximate τ_d_/τ_b_ ratio of 1, showing that for each docking concentration, there is an optimum counting area. Since the τ_b_ is inversely proportional to *k*_off_, τ_d_ is the dominant factor in tuning the τ_d_/τ_b_ ratio for optimum counting precision. As shown in eq 1, the τ_d_ can be altered by docking concentration (*n*), counting area (*A*), and imager concentration (*c*_i_). These three parameters can be tuned independently to achieve the desired τ_d_/τ_b_ ratio.

Additionally, the counting precision can be further increased by longer acquisition times (*t*) by a factor of , owing to the underlying Poisson statistics. This indicates that precision is a tradeoff between time and all the aforementioned parameters in eq 1. Recently, there have been efforts in improving the DNA-PAINT imaging speed.^27,31−33^ All these parameters influence qPAINT precision and accuracy by affecting the amount of events sampled. However, there is no benchmark on how many events should be collected for optimal precision and accuracy. Using simulated data, we correlated the number of events with the counting precision and accuracy (Figure S3b). We observed that requirements for an optimal accuracy are lower (accuracy saturates from 30 events) than those for an optimal precision, which reaches a plateau from 100 events. Here, we present the benchmark for accurate and precise qPAINT measurements with an acquisition of 100 times (τ_d_ + τ_b_) to achieve around 100 events, at a τ_d_/τ_b_ ratio of 1 for optimum imaging speed.

Kinetic counting offers precise and accurate counting through all range of densities if the right conditions are met. However, for lower density samples, direct counting is still less disturbed by non-specific interactions since we can use the spatial information to filter them out. Moreover, direct counting retrieves the exact position of each molecule giving a more accurate visualization of the distribution. Although kinetic counting cannot observe the nano-distribution of all molecules, it can observe fluctuations of density across the surface as shown in the density map for a 30 nM docking concentration coverage (Figure 3d). By optimizing the counting area based on Figure 3c, the number of dockings was counted precisely, revealing notable density fluctuations across the functionalized coverslip. The heterogeneity of surface functionalization may have critical implications in the behavior of these synthetic surfaces with their environment.

Quantifying the density of proteins and receptors on the membranes of cells is of foremost importance for biological and clinical research,^6,34,35^ and the importance of super-resolution imaging in this field is increasing. The applicability of DNA-PAINT to cell samples presents further challenges since the complexity of the cell membrane composition and the hydrophobicity of the lipid bilayer induce increased non-specific interactions compared to previously studied synthetic surfaces. Moreover, membranes cannot be designed with antifouling properties as synthetic surfaces; therefore, there are certain aspects that need to be considered and a further adaptation of the previously described workflow is presented. Briefly, three aspects are discussed in this work in the context of DNA-PAINT in cell membranes: the kinetic filtering of the non-specific interactions by (i) bright times (τ_b_) and (ii) dark times (τ_d_) and (iii) the influence of imager’s organic dye on the number of non-specific interactions.

**Figure 4:**
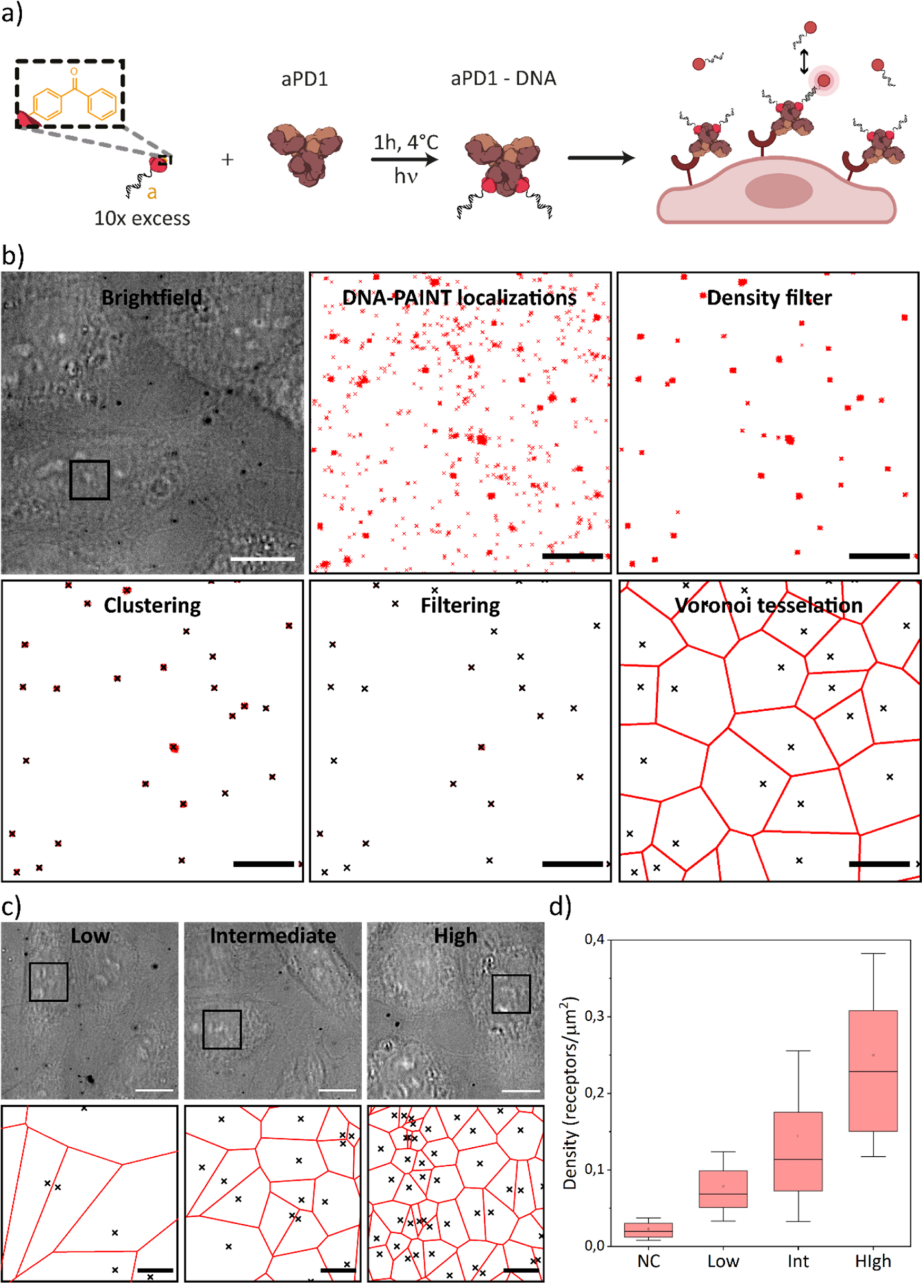
Direct counting of PD1 receptors on CHO cells. (a) Scheme of antibody labeling and cell membrane receptor imaging. (b) Workflow of data analysis: ROI selection on brightfield image (b1), reconstructed PAINT localizations (b2), density filter to remove sparse unspecific binding (b3), clustering (b4), time-trace cluster filtering (b5), and Voronoi tessellation to display density and distribution (b6). (c) Brightfield (c1-3) and final receptor images (c4-6) of PD1 low-, intermediate-, and high-expressing cell lines, respectively. (d) Density quantification of PD1 receptors on 50 cells per cell line. Negative control (NC) is performed on CHO cells not transfected with the PD1 receptor. Boxplot: box center represents the median; box limits are the 25–75 percentiles; the dot is the mean; and whiskers are the σ. Scale bar: bright field images (10 μm); PAINT images (a) (1 μm) and (b) (2 μm). Kinetic counting of EGF receptors on A-431 cells.

Here, we present two cases to which the previous approaches discussed in this work (direct and kinetic counting) would be applied. First, a panel of three transfected CHO cell lines stably expressing low levels of the PD-1 receptor would be analyzed by direct counting, and second, the high expression of EGFR in A-431 cells would be quantified by kinetic approach (qPAINT). To label target receptors on these cells, antibodies coupled with docking strands were used. The standard strategy to label antibodies is based on maleimide click chemistry,^23^ which would result in a distribution of the number of docking strands on each antibody and an arbitrary position of those strands on the antibody. This would from one side reduce the quantification precision and could interfere with the binding of the antibody if the docking strand attaches to the antigen binding site. Therefore, using a site-specific procedure described recently by Cremers et al.,^36^ selectively coupling two DNA docking strands to each antibody was performed. This is achieved by coupling the maleimide docking sequence to the *N*-terminal cysteine of protein G, which specifically binds to two sites on the antibody Fc region. The protein G is then photocross-linked to the antibody to ensure it remains bound. In this way, a controlled number and narrow distribution of docking strands per antibody is achieved, without interfering with the recognition domain (Figure 4a).

When imaging highly expressing EGFR A-431 cells with the 9-mer complementary sequence 1 and the non-complementary sequence 2 (Table 1), the density of binding events on the membrane is similar, meaning that >90% of events are non-specific interactions (Figure S5). Moreover, these events have similar binding times, therefore making them difficult to distinguish. However, switching from a 9-mer DNA interaction to a 10-mer DNA pair increases τ_b_ of the specific interactions compared to the non-specific ones due to a decrease in *k*_off_ (while *k*_on_ remains similar).^24^ Using a longer exposure time (150 or 300 instead of 90 milliseconds), most of the short-lived non-specific interactions were not detected due to their lower brightness or only appear as single-frame events. This drastically reduces the contribution of non-specific interactions, which is reduced below 2% by filtering-out these single-frame events (Figure S5).

However, when imaging low-expressing PD-1 CHO cells, the fraction of specific interactions is lower than that with the A-431 cells (fewer receptors), therefore increasing the influence of the non-specific fraction. This is mainly caused by the increase in the imaging time (30 to 60 min) and imager concentration (0.1 to 1 nM) in low-density samples required to identify single receptors, which accumulate more non-specific interactions. Since for low-density samples, we use direct counting analysis (clustering of localizations), we can use the spatial information to filter out the non-specific events. We observed that there are two types of non-specific interactions: (i) sparse one-frame events and (ii) clustered localizations that spatially resemble clusters of specific binding interactions. The former can be filtered out by the density difference to the specific dense spots using a density filter and during the clustering algorithm process (Figure 4b). The latter would be recognized as clusters and would require further filtering to remove these artifacts. Although having similar densities to the specific clusters, they are kinetically different (Figure S6b). In the dark times (τ_d_), these two types of clusters can be used to identify the artifacts and filter them out. With the help of simulations of specific DNA binding, we can determine that more than 99.99% of specific clusters would comprise at least 50% of imaging time between the first and last binding events (Figure S6c), removing the short-lived artifacts (Figure 4b). Another important parameter to consider is the nature of the organic dye on the imager sequence, which proved to be crucial since its chemical properties have a profound influence on the non-specific interactions with lipid bilayers.^3^ In this work, two different red dyes were utilized: ATTO647N (quantum yield of 65% but higher affinity for lipid bilayers) for samples where non-specific interactions were lower and there is a need for a brighter dye (imaging the apical membrane of A-431 cells in HiLo) and ATTO655 (quantum yield of 30%, but low affinity for lipid bilayers) for samples where non-specific interactions were more influential but brightness of the dye is not a limiting factor (imaging the basal membrane of CHO in TIRF).

### Direct Counting of PD1 Receptors on CHO Cells

The expression level of receptors on the membrane of cells has a direct impact on their behavior, and a correlation is found in diseases such as cancer, where receptors are used as biomarkers for diagnosis and prognosis.^37−40^ For instance, Nerreter et al.^19^ recently demonstrated that localization microscopy can detect low expression levels on cells, not detectable by flow cytometry, that are relevant for CAR-T cell therapy. This opens the door to measuring smaller and earlier changes in receptor expression to offer a better diagnosis. To demonstrate the great sensitivity of DNA-PAINT to quantify the density of receptors on the membrane of fixed cells, engineered PD1-expressing CHO cells were selected as a model. This provides cells with sparse receptors at three controlled levels (low, intermediate, and high) and provides a good negative control with the wild-type CHO cells that do not express this receptor. As explained for the synthetic surfaces, with DNA-PAINT, low densities of receptors can be quantified with high accuracy through a clustering algorithm and non-specific interactions can be filtered out during the analysis process. This is particularly important on cell membranes since there is a higher degree of non-specific interactions, not only from the labeled imager sequences but also from impurities that may bind to the surface.^2^

DNA-PAINT images were analyzed with the custom Matlab clustering script described previously. To tackle the challenges that imaging cell membranes present, a couple of steps were added in the analysis. First, a spatial density filter is applied to the DNA-PAINT localizations prior to clustering to remove most of the sparse non-specific localizations (Figure 4b). This does not affect receptors since they appear as a tightly packed group of high-density localizations due to repeated interactions with the DNA docking strands on the antibodies. After cluster identification, clusters that are caused by repeated non-specific interactions are filtered out by discarding clusters for which the first and last event comprehend less than 50% of the imaging time (Figure S6). By doing this, artifacts are removed and it is possible to obtain the accurate density and distribution of receptors as shown in the tessellation (Figure 4b).

Figure 4c shows a representative reconstruction of the distribution of receptors for the three different cell lines. The receptors appear disperse but homogeneously distributed. From these examples (Figure 4c), the difference in receptor density between the 3 cell lines is noticeable. Plotting (Figure 4d) the densities of 50 individual cells for each one of the cell lines, the average of each population can be seen: Low = 0.08 receptors/μm^2^, Int = 0.14 receptors/μm^2^, and High = 0.25 receptors/μm^2^. This is in accordance with flow cytometry measurements on these cells published by Cremers et al.^41^ From this, we can conclude that the approach and data analysis procedure are not sensitive to unspecific interactions and artifacts evidenced by the variation in the counted number of receptors between the control and the expressing cell lines (Figure 4d NC), even at a low receptor expression. This leads to high sensitivity and opens the door to detect low levels of expression due to the high specificity of the method. This is of significant importance to future studies since it is a clear advantage compared to the benchmark techniques employed to measure receptor densities, such as flow cytometry,^42−44^ immunohistochemistry,^45−47^ ELISA,^48,49^ or fluorescence in situ hybridization.^50,51^ The impact of the ability to quantify low expression levels has already been demonstrated for cancer diagnosis^19^ but is likely to be relevant for a wide range of diseases.

The combination of the high sensitivity (down to the single-molecule level) and the high accuracy and precision of this approach (Figures 2 and S4) enables the distinction between the three cell populations (Figure 4d). However, there is a high variability within each one of these populations. Assuming a minimal impact on variance from methodological errors (observing individual receptors), the coefficient of variation of 60–80% within each cell population is mostly related to biological factors such as transfected gene copy number or cell cycle stage. This is important since in many cancer types, there is a high heterogeneity within the tumor, which can cause resistance to certain therapeutics that only target a subset of the cells. Measuring this heterogeneity would provide guidance in the improvement of cancer diagnostics and subsequent treatment development.

**Figure 5:**
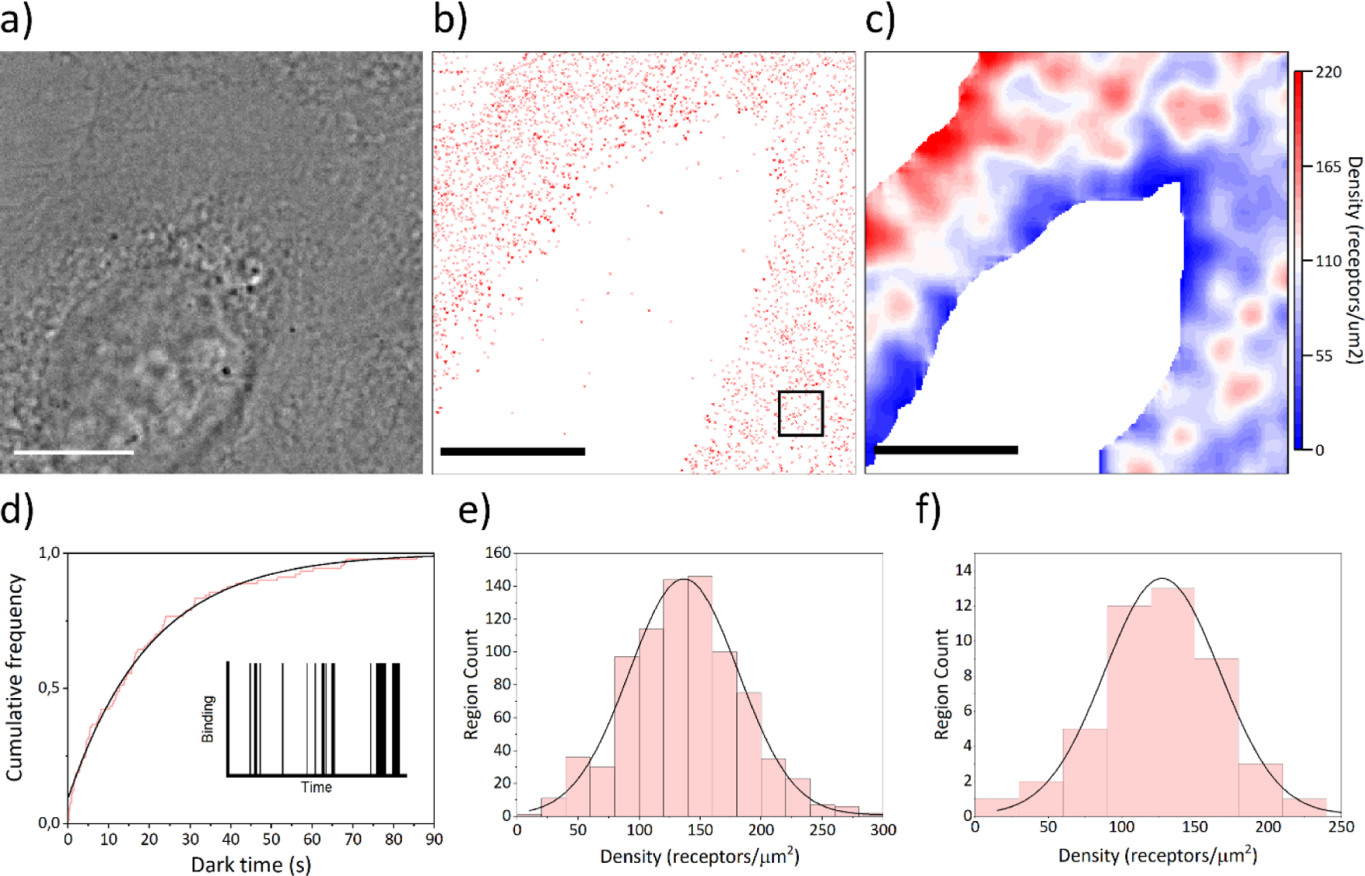
Kinetic counting of EGFR on an A-431 cell. Workflow of receptor quantification: (a) bright field image, (b) PAINT binding events (red dots), and ROI selection (black box, 1 μm^2^) and (c) final density analysis by qPAINT and smoothing. (d) Example of a CDF of a time trace (red) and exponential fitting (black). (e) Distribution of all the regions analyzed across all cells and Gaussian fitting. (f) Distribution of the average density value per cell and Gaussian fitting. Scale bar: 5 μm. Optimized workflow for DNA-PAINT quantification of surface-bound molecules.

There are cases in nature where the expression level of receptors is significantly higher than those previously analyzed in this work. For instance, many receptors are overexpressed in cancer to promote faster and uncontrolled growth of the tumor. Although this does not represent a challenge in terms of sensitivity, many techniques are not capable of quantifying the receptor density because it is not trivial to convert a fluorescence intensity to a local density. In addition, most techniques do not reveal inter-cell variability. With the kinetic counting approach that is outlined above for synthetic surfaces, it is possible to quantify the density and distribution of highly packed receptors on cell membranes (Figure 3). As a model, labeled EGFR on fixed A-431 epidermal carcinoma cells was investigated, as they are known to have a high expression of this receptor.^52^ Since it is observable that these cells exhibit an extremely low density of receptors on the basal membrane, the apical part of the cell was focused on. Figure 5a–c shows the brightfield image (a), DNA-PAINT events (b), and qPAINT analysis (c) to calculate the density and visualize the distribution of EGFR on a single A-431 cell. The cells were imaged using *c*_i_ = 10 pM of the ATTO647N imager for 30 min to obtain thousands of events per cell, in this way matching the previously set goal of 100 events per counting area (black square in Figure 5b) to achieve the optimal precision and accuracy (Figure S4). To calculate the density of receptors using eq 1, the calibrated value of *k*_on_ on DNA-functionalized PD1 antibodies from previous experiments was taken (Figure S6d), together with the average dark time obtained from the cumulative distribution of events in a counting area (Figure 5d).

Qualitatively, it is possible to observe the distribution of receptors on a single cell basis. In this case, there seems to be a higher concentration of receptors close to the edge of the cell membrane (right side of Figure 5c) and more random fluctuations of the density across the membrane. The lower density observed on the inner part of the cell is a result of losing events due to the membrane being out of focus (the apical membrane gets higher where the nucleus is). The quantitative results of density from qPAINT analysis of different areas of cells and individual cell densities are shown in Figure 5e,f, respectively. From the 50 A-431 cells imaged, the EGFR are widely distributed between (Figure 5f) as well as within cells (Figure 5e), with an average around 130 receptors/μm^2^. The total variability observed among cells (σ_N_total_^2^ = 1938.6) in the different sources can be attributed to (i) σ_N_qPAINT_^2^ from the limited number of events (σ_N_qPAINT_^2^ = 409.5, Figure S4b), (ii) σ_N_poisson_^2^ from the natural Poisson distribution of receptors (σ_N_poisson_^2^ = 126.5), and (iii) σ_N_bio_^2^ from other biological factors. This yields σ_N_bio_^2^ = 1402.6, which is notably the highest contributor to the main variability. It implies that variability due to error sources is low, confirming that the observed variability comes from a true biological difference between cells. Such quantifications are crucial to discern and understand heterogeneity in cancer for better diagnosis and treatment.

**Figure 6:**
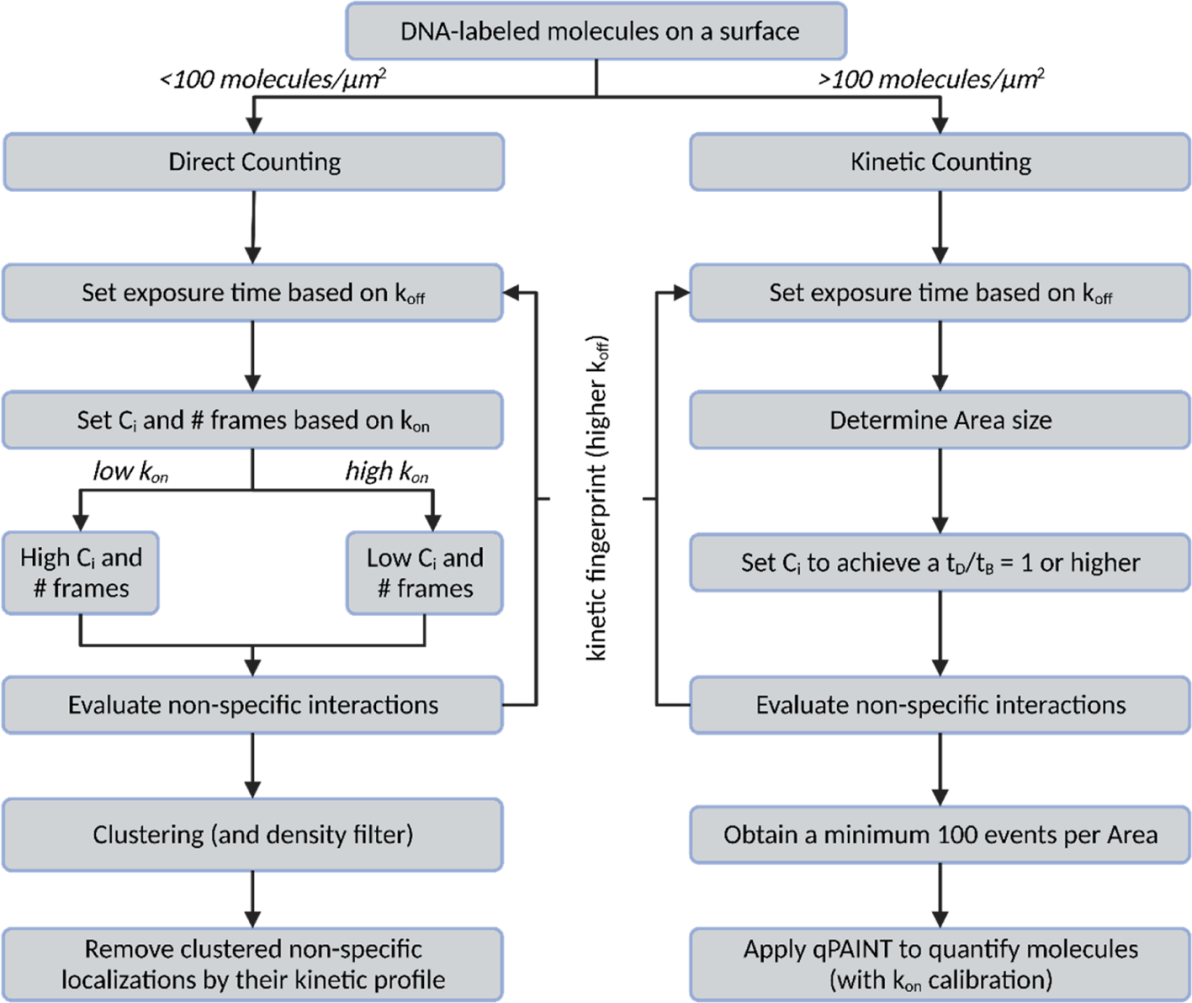
Optimized workflow for quantification of molecule density and distribution on surfaces.

Throughout this work, we have standardized many aspects of the quantification of molecules on surfaces using DNA-PAINT. In Figure 6, we have summarized the main findings in a flowchart to guide set up of new DNA-PAINT quantification experiments. We compared two main approaches on the basis of molecular density on surfaces. Direct counting allowed for the exact localization of molecules, density quantification, and the visualization of spatial distribution. It requires a clustering algorithm to identify the position of molecules but it uses the spatial information to filter out non-specific interactions. However, it is limited to low density samples (<100 molecules/μm^2^) since at higher densities, it is not feasible to discriminate between different molecules. One the other hand, kinetic counting exploits the kinetic information of DNA–DNA interactions to quantify molecules even at high densities, bringing a broader dynamic range than direct counting. However, it requires a prior calibration of k_on_ and it does not provide the nano-scale distribution of molecules.

## Conclusions

A standardization of the DNA-PAINT workflow is introduced to quantify the number and distribution of molecules in two-dimensional surfaces with high precision and accuracy. With the help of experimental and simulated data, the best quantification strategy was determined based on the molecular density, providing single-molecule sensitivity on a large dynamic range. Then, depending on the selected quantification method, the optimal conditions for faster, accurate, and precise counting were calculated. This is done by tackling the specific hurdles of DNA-PAINT imaging on surfaces and using kinetic and spatial filtering to reduce the impact of the non-specific interactions, yielding single-molecule sensitivity and improving counting precision and accuracy. We provided a detailed description of the quantification performance to ensure the robustness of the workflow.

The foreseen application of this approach is to enable more detailed investigations and thus understanding of the design of synthetic surfaces (i.e.*,* biosensors) and the role of receptors and their distribution in disease and treatment development. Most importantly, the approach provides information on the distribution and heterogeneity of surface-bound receptors in fixed cells. This is key since the organization of the molecules on the surface determines how they interact with the environment, which can modify the behavior of a biosensor. Moreover, it can bring information about the variety of cells in a tumor and better predict how they would reply to a certain treatment or help find the right combination of therapies that would target all cell subsets. Although it is an approach employing wide-field imaging, fine-tuning of the single-molecule association, dissociation kinetics,^31,33^ and automatization of processes^53^ will reduce the acquisition time and further improve the throughput of this approach.

## Materials and Methods

### Materials

DMEM/F12 medium (HEPES, no phenol red), FBS, penicillin/streptomycin, biotinylated BSA, and streptavidin were purchased from Thermo Fisher Scientific (Massachusetts, US). Geneticin sulfate was purchased from Capricorn Scientific (Ebsdorfergrund, Germany). Culture 6 channel μ-Slide #1.5 glass bottom was purchased from Ibidi (Gräfelfing, Germany). Polystyrene nanoparticles of 340 nm were purchased from Spherotech (Illinois, US). Gold nanoparticles of 80 nm were purchased from Sigma-Aldrich (Missouri, US).

### DNA-PAINT Sequences

Oligonucleotides were purchased lyophilized and were resuspended in TE buffer upon arrival. Concentration was measured with a NanoDrop 2000 (ThermoFisher Scientific), and aliquots were stored in the freezer.

### Synthetic Surface Preparation for DNA-PAINT Imaging

Coverslips #1.5 were rinsed with ethanol and milli-Q water and then placed into an ultrasound bath with fresh ethanol for 15 min. Afterward, they were rinsed extensively with milli-Q and dried with nitrogen. For the sample preparation, a coverslip (24 × 24 mm) and glass slide (25 × 75 mm) were sandwiched by two strips of double-sided tape to form a capillary chamber with an inner volume of ∼30 μL.

To prepare the glass surface, 30 μL of biotin-labeled bovine albumin (fixed at 0.1 mg/mL) dissolved in buffer A (10 mM TRIS–HCL, 50 nM NaCl, pH 8.0) was flown into the chamber and incubated for 1 h (all incubation steps are done in a humidity box to prevent drying). The unbound albumin was washed away by 200 μL of buffer A. Then, 30 μL of streptavidin (fixed at 0.1 mg/mL) was flown through the chamber and allowed to bind for 15 min. After washing away the excess streptavidin with 200 uμL of buffer A and subsequently with 200 μL of buffer B (5 mM TRIS–HCL, 10 mM MgCl2, 1 mM EDTA, pH 8.0) for buffer exchange, 30 μL of biotin-docking DNA was added and incubated for 10 min (docking concentration ranged from 0.2 to 1000 nM). To remove the unbound docking strands, the chamber was washed with 200 μL of buffer B. Later, Cy3-labeled polystyrene nanoparticles were used as fiducial markers for subsequent drift correction, incubated for 5 min, and then washed with 200 μL of buffer B. Freshly prepared ATTO655 imager strand 1 in buffer B was flown before imaging. Lastly, the chambers were sealed at both ends. BSA, streptavidin, and ATTO655 imager concentration remained fixed for all docking conditions.

### Cell Culture and Immunostaining

In this work CHO–K1 (ATCC CCL-61), CHO-PD1 (monoclonal CHO–K1 cells stably expressing low, intermediate, and high levels of PD1, kindly provided by Aduro Biotech), and A-431 (CRL-1555) cells are used. All CHO cells are grown in DMEM/F-12 medium supplemented with 10% FBS, 100 units/mL penicillin, and 100 μg/mL streptomycin, and CHO-PD1 lines are additionally supplemented with 0.6 mg/mL of G-418. A-431 cells are grown in DMEM medium supplemented with 10% FBS, 100 units/mL penicillin, and 100 μg/mL streptomycin. Antibodies (anti-PD1 and cetuximab) were functionalized with DNA sequences as described by Cremers, et. al.^36^

For preparation for imaging, cells were cultured overnight in an 8-channel Ibidi slide to achieve a confluence of 70–90%. Cells were first let to equilibrate at room temperature for 5 min, following 5 min equilibration at 4 °C. Before antibody incubation, cells were washed with chilled DMEM containing 3% BSA. Cells were then incubated in DMEM containing 3% BSA and 1 μg/mL of DNA-modified antibodies for 45 min at 4 °C. Afterward, three washing steps of 5 min with PBS were performed to remove the unbound antibody, followed by 10 min fixation with 3.7% PFA and 0.25% glutaraldehyde in PBS at room temperature (from this point, all steps are carried out at room temperature). Cells are again washed with PBS three times for 5 min and incubated with glycine 0.1 M for 10 min to block any remaining reactivity of the fixative. Lastly, after three more 5 min PBS washing steps, cells are incubated with 80 nm gold nanoparticles at 1:5 dilution for 10 min. Unbound gold nanoparticles are removed by three washings with PBS before samples are stored sealed in the fridge until imaging.

### Optical Setup and Image Acquisition

DNA-PAINT images were obtained in an Oxford Nanoimager microscope (ONI, Oxford, UK). The sample was illuminated using total internal reflection fluorescence (TIRF), and fluorescence was recorded using a 100×, 1.4 NA oil immersion objective, passed through a beam splitter to obtain a green and a red channel. Images were acquired onto a 427 × 520 pixel region (pixel size 0.117 μm) of a sCMOS camera. Images were reconstructed using the ONI Nimos software in order to identify and fit the point spread functions and obtain the super-resolved position of the target molecule.

DNA-PAINT images of the synthetic surfaces were acquired with an exposure time of 90 milliseconds under 30 mW of a 640 nm laser for 22,000 frames. Additionally, Cy3-labeled polystyrene drift correction particles were illuminated one every hundred frames with 5 mW of a 532 nm laser and recorded simultaneously on a second channel, created by the beam splitter that separates the light onto two separate parts of the camera. Drift correction was performed in the Nimos software using the positions recorded of the Cy3B-labeled particles. The concentration of the ATTO655 imager was set to 5 nM for all glass-experiments.

DNA-PAINT images of PD1 receptors on CHO cells were acquired with an exposure time of 300 milliseconds under 30 mW of a 640 nm laser for 12,000 frames. Images of EGFR on A-431 cells were acquired with an exposure time of 150 milliseconds under 30 mW of a 640 nm laser for 12,000 frames. Drift correction is performed with 80 nm gold nanoparticles emitting on the same channel as the DNA-PAINT imagers. Localizations are linked together using the single-particle tacking tool in Nimos software to identify the non-blinking spots (trajectories with 12,000 frames), corresponding to the gold nanoparticle. Later, a custom Matlab script is used to correct the DNA-PAINT localization positions with the information of gold nanoparticle displacement. The imager concentrations (ATTO655 and ATTO647N) were set to 1 nM for CHO cells (direct counting) and 0.1 nM for A-431 cells (kinetic counting).

### Direct Counting Analysis

To identify sparse molecules in DNA-PAINT images, a custom Matlab algorithm was used. Briefly, a mean-shift clustering algorithm is employed to identify multiple events spots, corresponding to specific binding to the target molecule. Mean-shift clustering is a non-parametric analysis that identifies local density maxima (dense spots) by shifting a window toward the density maximum inside that area, until convergence. This method is selected because it is based on the identification of dense spots with a specific circular shape. The recorded data on cell membranes is first treated with a density filter (minimum 5 localization in a 25 nm radius circle) since there is a higher non-specific interaction in hydrophobic cell membranes. Next, a mean-shift clustering (bandwidth of 50 nm and minimum 10 localizations per cluster) is applied to identify the positions of the target molecules and discriminate the sparse non-specific localizations. Lastly, non-specific localizations are also found forming clusters in cell membranes; therefore, localizations are merged into events (maximum frame gap of 10 frames) in order to obtain time traces. Non-specific clusters do not follow the expected binding kinetics time trace, and events are concentrated over a short period of time. These are filtered out by removing the clusters that do not last for more than 50% of the imaging time.

### Kinetic Counting Analysis

Analysis of DNA-PAINT images of densely packed surfaces was done using a custom Matlab algorithm. First, localizations were merged into binding events (maximum frame gap of 3 frames and maximum distance between frames of 50 nm) in order to identify time traces. Short events of only one frame, and events with less than 1500 photons are discarded for images acquired with the ATTO647N imager. A desired area is selected, and the dark times between events are extracted, and the CDF is fitted with eq 2 to obtain the mean value (τ_d_). This value is then used in eq 1 to calculate the number of target molecules (*n*) in a certain area (*A*), considering the concentration of the imager (*c*_i_) and the binding rate constant *k*_on_.
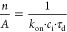
1
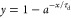
2

In order to create the density maps in Figures 3d and 5c, a small-sized ROI (9 × 9 camera pixels) is selected. Using a Matlab algorithm, it is iterated through the entire image randomly using the camera pixels as binning. At every position, the density of molecules is calculated with qPAINT inside the ROI and noted in each of the pixels inside the ROI. Once every pixel has been iterated 100 times, the different density values are averaged and plotted.

### Logistic Fit

Quantification of the molecular counting range (lower, higher bounds, and mid-point values) of both methods (direct and kinetic) was performed in OriginLab using a logistic dose response equation, where *A*1, *A*2, *X*_0_, and *p* correspond to the theoretical response to zero concentration, infinite concentration, mid-range concentration, and slope factor, respectively.
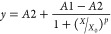
3

### DNA-PAINT Simulations

DNA-PAINT simulations were performed in MATLAB. Simulations of sparse molecules for direct counting approach (Figure S3a) were performed by randomly simulating the positions of molecules at specified densities. Then, following a gaussian distribution centered on each molecule position, single-molecule events were generated using the average experimental precision (σ = 50 nm). On the other hand, dark times for kinetic counting approach (Figure S3b) were generated using an exponential decay distribution with an average τ_D_ of 401 s (derived from the experimental *k*_on_ of 2.49 × 10^–6^ M^–1^s^–1^ and an imager concentration of 1 nM).

## Supporting Information Available

Typical field of view of single-molecule fluorescence events, histograms of dwell times (dark-and-bright) of binding events, simulation of the direct and kinetic counting performance, control experiments, cumulative distribution functions and calibration, time traces, and number of events on cell membranes (PDF)
