# Supplementary material for: Precision and Accuracy of Receptor Quantification on Synthetic and Biological Surfaces Using DNA-PAINT
Source: ACS Sens. 2023 Jan 19;8(1):80–93. doi: 10.1021/acssensors.2c01736 (PMC9887648; doi:10.1021/acssensors.2c01736)
Supplement: Supplementary file 1 — se2c01736_si_001.pdf [file se2c01736_si_001.pdf]

Precision and accuracy of receptor quantification on synthetic and biological surfaces using DNA-PAINT

Roger Riera[1]†, Emmanouil Archontakis[1]†, Glenn Cremers[2,3], Tom de Greef[2,3,4], Peter Zijlstra[5*], Lorenzo Albertazzi[1,6*]

[1] Department of Biomedical Engineering, Institute for Complex Molecular Systems (ICMS), Eindhoven University of Technology, P.O. Box 513, 5600 MB Eindhoven, Netherlands

[2] Laboratory of Chemical Biology and Institute for Complex Molecular Systems, Eindhoven University of Technology, P.O. Box 513, 5600 MB Eindhoven, The Netherlands

[3] Computational Biology Group, Department of Biomedical Engineering, Eindhoven University of Technology, P.O. Box 513, 5600 MB Eindhoven, The Netherlands

[4] Institute for Molecules and Materials, Radboud University, Heyendaalseweg 135, 6525 AJ Nijmegen, The Netherlands

[5] Department of Applied Physics and Institute for Complex Molecular Systems, Eindhoven University of Technology, P.O. Box 513, 5600 MB Eindhoven, The Netherlands

[6] Nanoscopy for Nanomedicine, Institute for Bioengineering of Catalonia, 08028 Barcelona, Spain

† These authors have contributed equally.

Supplementary Figures

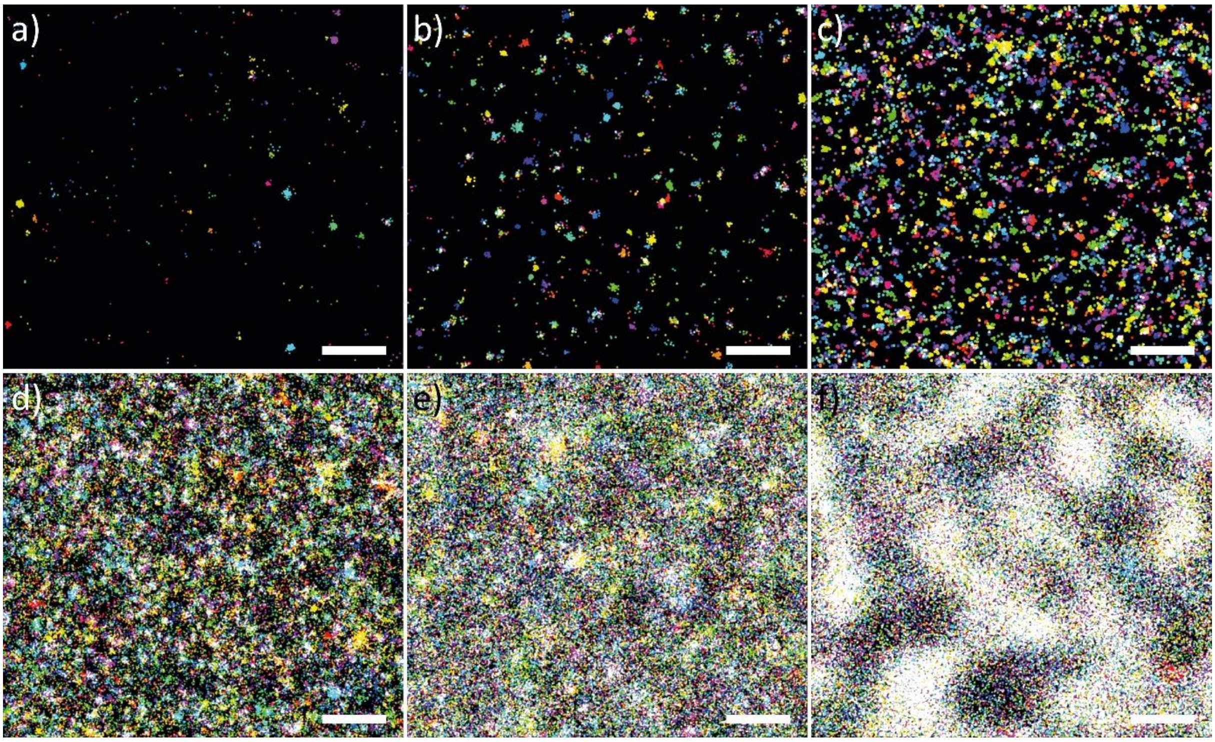

Figure S1. (a-f) DNA-PAINT reconstruction maps; 0.2 nM, 2 nM, 5 nM, 20 nM, 60 nM, 500 nM docking strand concentrations, respectively. Imager concentration: 5 nM. Exposure time: 90 ms. Frames: 22k. Scale bar: 1 µm.

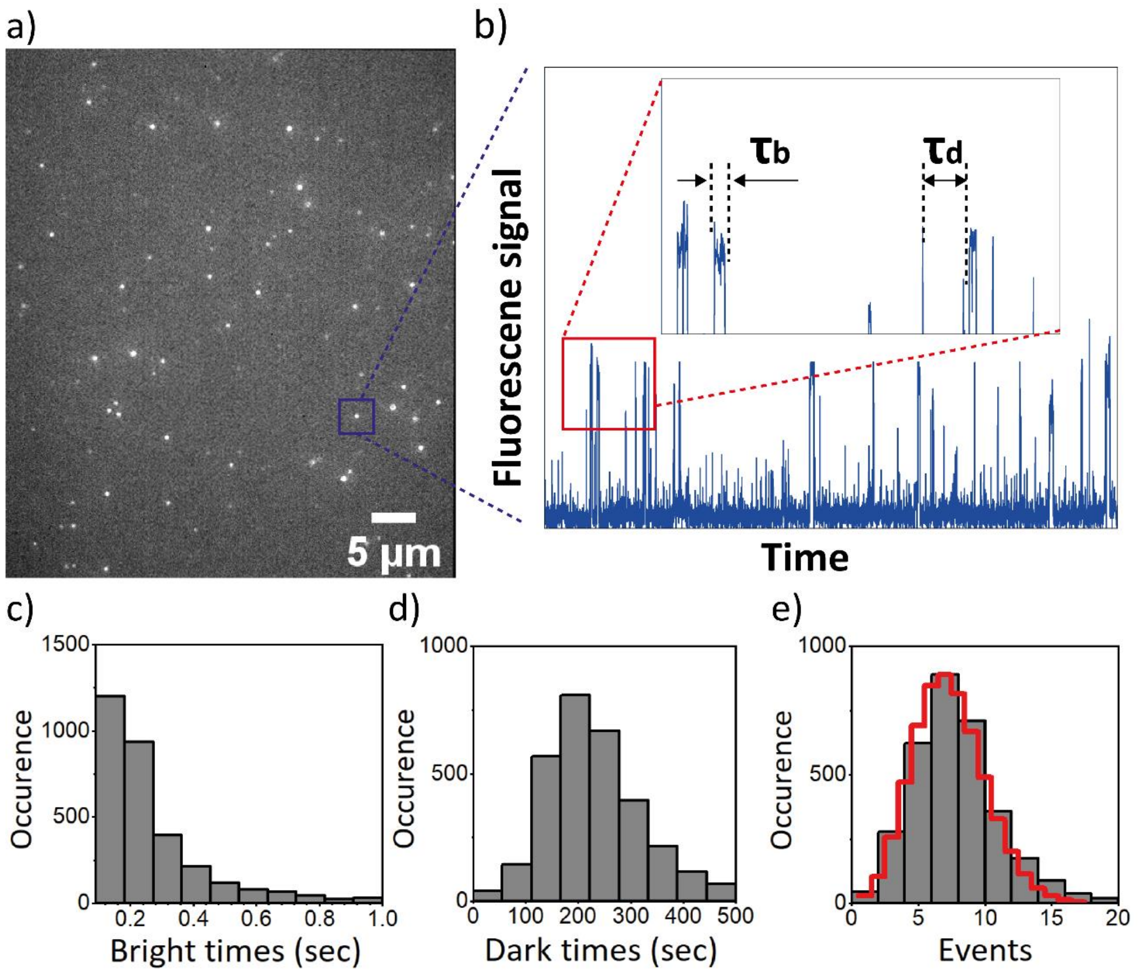

Figure S2. (a) Typical field of view of a typical DNA-PAINT measurement TIRF mode, where each diffraction limited spot corresponds to a binding event on a docking strand. (b) Each fluorescence binding event causes a fluorescent signal in time and can be depicted in a single time trace. (c-d) Histograms of average bright (c) and dark time (d) per docking strand extracted from the time traces. (e) Histogram of the total number of events per docking strand, where clusters correspond to single dockings following a Poisson distribution (red line). Docking strand concentration: 10 nM.

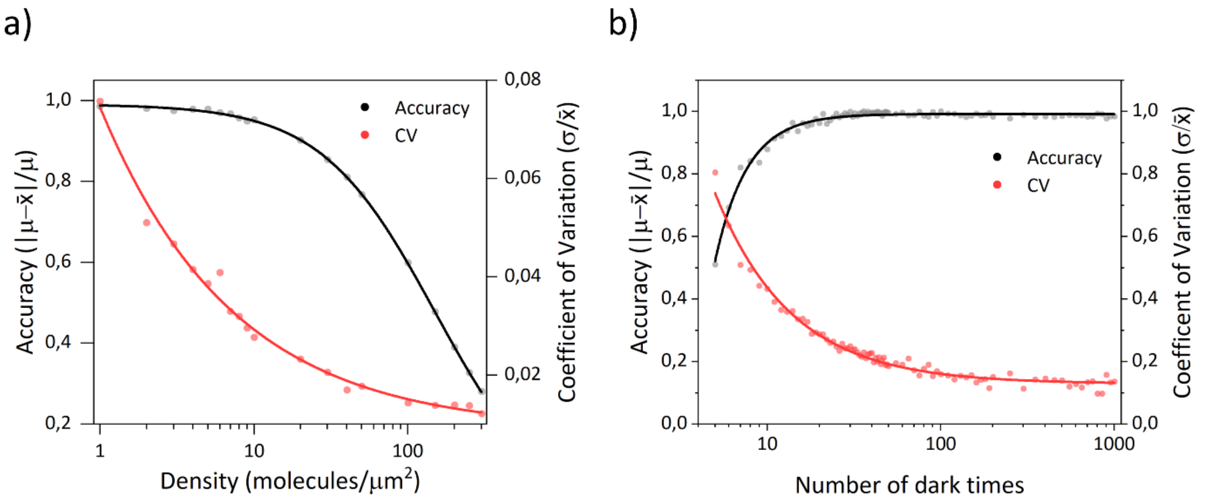

Figure S3. (a) Simulation of direct counting performance. Randomly generated DNA-PAINT localizations from individual molecules on a 2D surface at different densities are analyzed and the accuracy and the coefficient of variation are calculated. (b) Simulation on kinetic counting performance. Time traces with different number of events are generated to extract dark times. The accuracy and the coefficient of variation from the resulting value obtained from fitting these dark times are calculated.

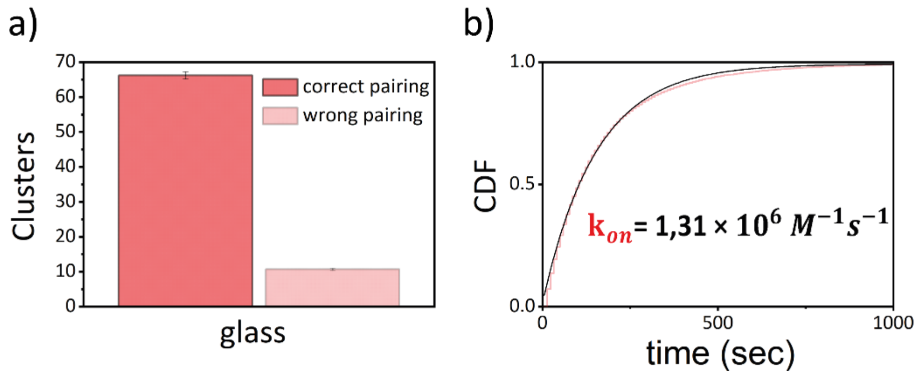

Figure S4. (a) Control experiments of 8 nM docking strands (Docking1) on the coverslip. Wrong pairing represents an oligonucleotide imager sequence (Imager2 – ATTO655) that was not complementary to the docking, in contrast to correct pairing, which represents an oligonucleotide imager sequence (Imager1 – ATTO655) complementary to the docking. In both cases clusters were detected and directly counted by the mean-shift algorithm. (b) Cumulative distribution function (CDF) of individual dark times obtained from clusters that represent a single docking strand.

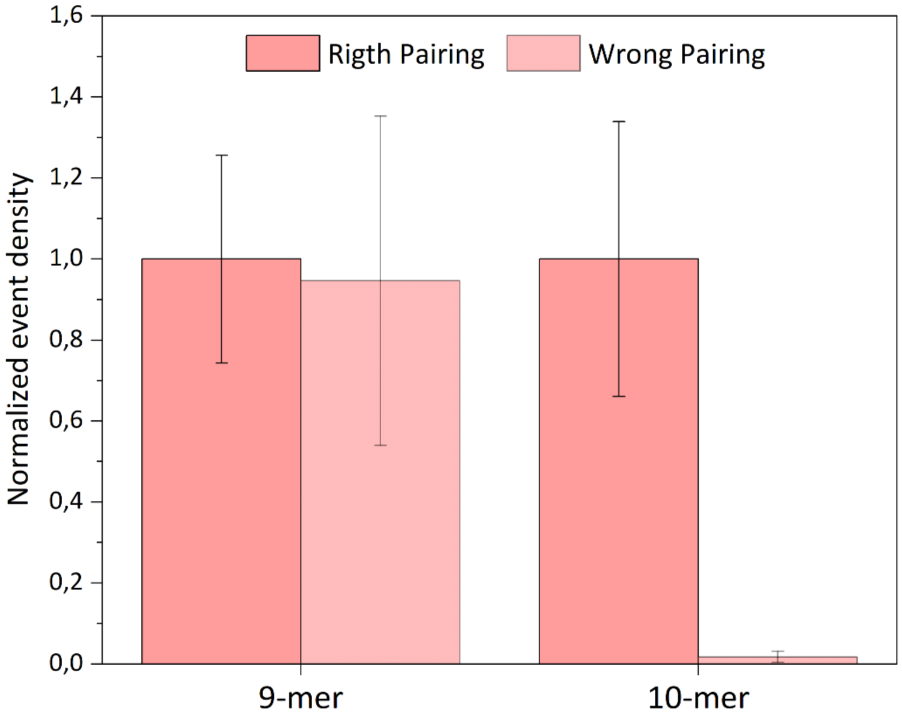

Figure S5. Density of events of ATTO647N 9mer and 10mer imagers on docking-cetuximab labelled A-431 cell membranes. Both right and wrong pairing are measured to determine level of unspecific interactions.

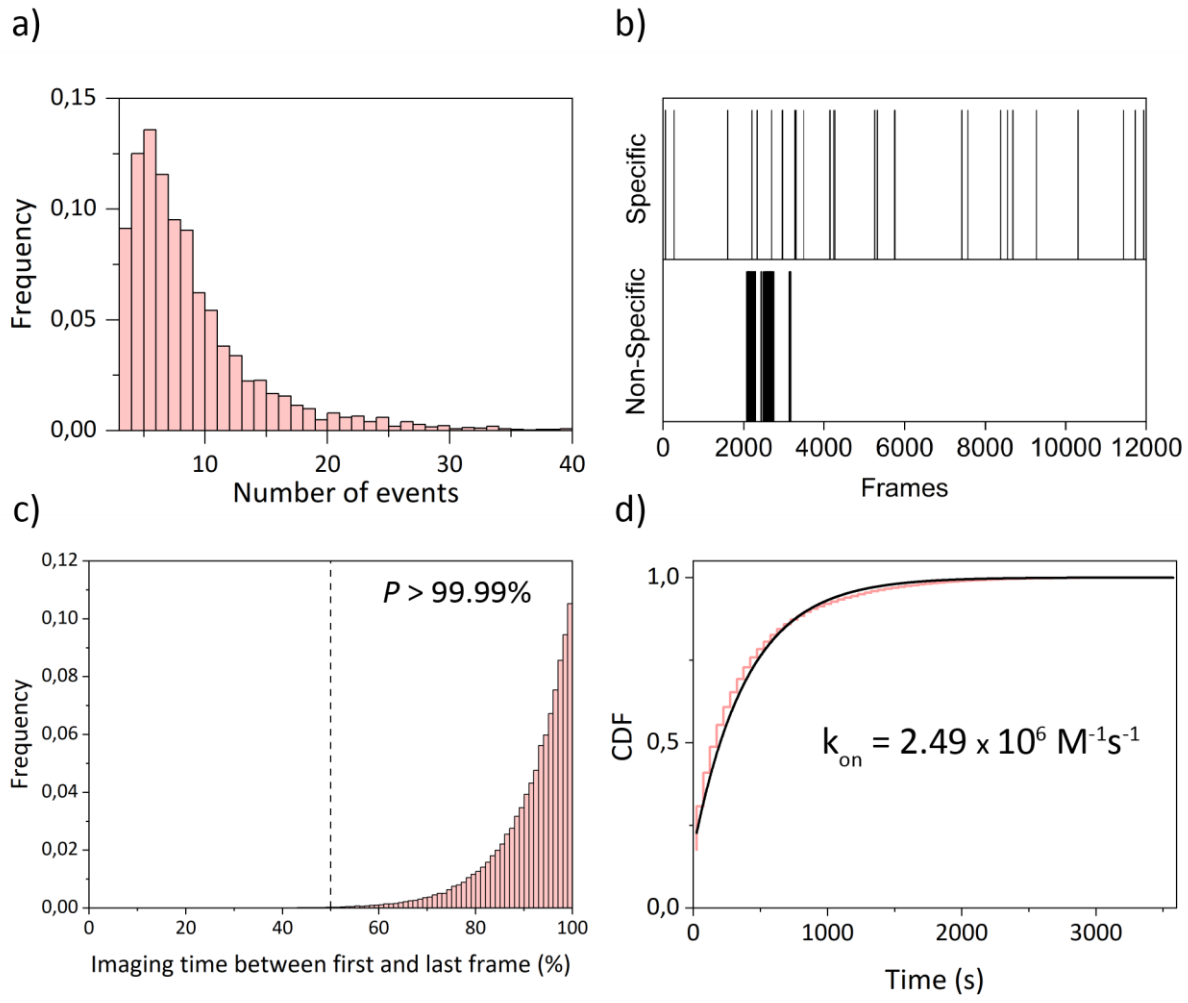

Figure S6. (a) Number of events of PD1 receptors clusters. (b) Time traces of PD1 clusters. Top: expected behavior of binding kinetics. Bottom: events appear concentrated in a region in time. (c) Simulation on percentage of imaging time between first and last frames of generated time traces. The threshold is set to 50% to keep clusters with a behavior of top b) and filter out cluster such as bottom b). (d) Combination of all dark times obtained from time traces of single PD1 receptors (single receptors, with 2 docking strands) in the CDF and fitting to obtain the k_{on} value of this DNA paint interaction.
